# Discordances in ER, PR, and HER2 between primary breast cancer and brain metastasis

**DOI:** 10.1007/s11060-017-2717-0

**Published:** 2017-12-19

**Authors:** Jaehag Jung, Seung Hoon Lee, Mira Park, Ji Hye Youn, Sang Hoon Shin, Ho Shin Gwak, Heon Yoo

**Affiliations:** 1 Department of Surgery, Eulji University Hospital, Daejeon, South Korea; 2 Department of Neurosurgery, Eulji University Hospital, Daejeon, South Korea; 3 Department of Preventive Medicine, Eulji University, Daejeon, South Korea; 4 Neurooncology Clinic, National Cancer Center Hospital, Goyang, South Korea

**Keywords:** Breast cancer, Brain metastasis, Discordance, Receptor conversion

## Abstract

When distant metastases are discovered, it is important to determine receptor profiles of these lesions through histologic examination. However, brain metastasis sites are difficult to reach to be routinely biopsied. The purpose of this study was to determine expression profiles of estrogen receptor (ER), progesterone receptor (PR), and human epidermal growth factor receptor 2 (HER2) in breast cancer brain metastasis (BCBM) and the existence of discordance between primary breast cancer and brain metastasis. A total of 37 patients who underwent craniotomies for metastatic brain tumors arising from breast cancer at National Cancer Center (NCC) of Korea between 2002 and 2014 were retrospectively reviewed. Clinicopathologic data were collected from electronic medical records. Receptor profiles of primary breast cancer and brain metastasis in each patient were identified. Data of ER, PR, and HER2 expression in brain metastasis were available in electronic medical records for 21 (56.8%) of 37 cases. Results of ER, PR, and HER2 expression were positive in 47.6, 42.9, and 38.1% of patients with brain metastasis, respectively. Receptor conversion occurred in 11 (52.4%) of 21 patients (for ER, 9.5%; for PR, 38.1%; for HER2, 23.8%). Overall survival was longer in patients with concordant receptor expression patterns between primary breast cancer and brain lesion compared to that in patients with discordant patterns. However, such difference was not statistically significant (discordant vs. concordant median survival: 19.2 versus 31.1 months, *p* = 0.181). Receptor conversion in BCBMs was observed in over 50% of Korean patients used in this study. HER2 conversion was observed in 23.8% of patients in this study. Therefore, if resistance to anti-HER2 treatment is suspected in patients with BCBM, biopsy is needed to determine receptor profiles of brain lesion.

## Introduction

Breast cancer is the second most common cancer in Korean women after thyroid cancer [1]. Early detection of this disease with screening and good standardized treatment has improved the prognoses of afflicted patients. However, approximately one-third of patients will develop distant metastases to the liver, bones, lungs, and brain. They eventually succumb to the disease.

Brain metastasis is the fourth most common metastatic site for patients with breast cancer. Its incidence rate ranges from 10 to 16% [2]. The rate of patients with breast cancer brain metastasis (BCBM) appears to be increased due to effective treatment of systemic disease and improved survival following diagnosis of primary cancer. In addition, improved imaging modalities have enabled early detection of subclinical diseases. Brain metastasis in patients with breast cancer has poor prognosis, with a median survival of 2–9 months despite treatment [3, 4]. Current standard treatment options for brain metastases include local treatments such as surgery, stereotactic radiosurgery, and whole-brain radiotherapy (WBRT). Chemotherapy and targeted therapy have been reported to be effective for approximately 30–40% of patients [5, 6]. Expressions of hormone receptors (HRs) and human epidermal growth factor receptor 2 (HER2) are critical for determining personalized treatment options for patients with breast cancer. However, BCBMs are generally treated with chemotherapy on the basis of the receptor profile of primary breast cancer BCBMs are generally treated with chemotherapy on the basis of the receptor profile of primary breast cancer due to limited access to metastatic brain lesions which leads to the inability to determine their receptor expression profiles.

Previous studies have reported that receptor statuses of breast cancer metastases might differ from those of primary tumors [4, 7–13]. These studies have suggested that estrogen receptor (ER) and progesterone receptor (PR) are frequently negative in distant metastases whereas HER2 is often positive. However, the majority of these studies have focused on the correlation between lymph node metastasis and primary cancer. Few studies have compared immunophenotypes of breast cancer to those of brain metastases in Korean patients.

Therefore, the objective of this study was to determine expression patterns of ER, PR, and HER2 in Korean patients with breast cancer who underwent craniotomy due to brain metastases. Expression patterns of ER, PR, and HER2 were also compared between primary breast cancer tissues and brain metastases.

## Methods

Records of consecutive patients who underwent craniotomy for metastatic brain tumors arising from breast cancer at the Neuro-oncology Clinic of the National Cancer Center of Korea between 2002 and 2014 were retrospectively reviewed. Thirty-seven consecutive patients who underwent craniotomy for BCBM were identified. Clinicopathologic data were collected from electronic medical records, including patient demographics, histological type, grade, tumor stage, biomarker status, date of diagnosis of breast cancer, subsequent brain metastases and craniotomy, number and locations of brain lesions, and survival. Patients with available data for ER, PR, and HER2 status were included in this study.

Status of ER and PR was determined by immunohistochemistry (IHC) reactivity. Samples with 1% or greater reactivity were defined as positive for both ER and PR receptors. HER2 overexpression was defined as a membrane staining score of 3+ (HER2+). Those with a score of 1+ and 0 were defined as HER2-negative (HER2−). Fluorescent in situ hybridization was performed when HER2 IHC scores were equivocal (2+).

The frequency of receptor expression in primary breast cancers and brain metastases was calculated. Overall survival, median survival, and 95% confidence intervals (CIs) were estimated with Kaplan–Meier analysis. Brain metastasis-free survival was also estimated. Subgroups were compared using both overall and pairwise log-rank tests. Statistical analysis was performed using SPSS version 20.0 (IBM-SPSS, Armonk, NY, USA). A *p* value of < 0.05 was considered statistically significant. This study was approved by the Institutional Review Board (IRB) of the National Cancer Center, Korea.

## Results

A total of 37 patients were included in this study. Their median age at initial diagnosis of breast cancer was 53.9 years (range 38–81). Patients’ characteristics are summarized in Table 1. The histologic type of most patients was invasive ductal carcinoma (81.1%, Table 1). Regarding TNM stage, those with stage II had the highest percentage (35.1%, Table 1). Regarding histologic grade, those with grade 3 had the highest percentage (54.1%, Table 1). Expressions of ER, PR, and HER2 in primary breast cancer were positive in 43.2, 35.1 and 51.4% of patients, respectively. Distributions of different biological subtypes of breast cancer were as follows: HR+/HER2− ,35.1%; HR+/HER2+, 8.1%; HR−/HER2+, 43.2%; and HR−/HER2−, 13.5%. Twenty-eight (75.7%) patients had solitary brain lesion at BCBM diagnosis (Table 1).

**Table 1:** Characteristics of patients included in this study

Feature	Grouping	N or value	%
Age	Mean	53.9	
	Range	38–81	
Histologic type	Invasive ductal carcinoma	30	81.1
	Others	4	10.8
	Unknown	3	8.1
Histologic grade	1	1	2.7
	2	8	21.6
	3	20	54.1
	Unknown	8	21.6
TNM stage	1	6	16.2
	2	13	35.1
	3	9	24.3
	4	1	2.7
	Unknown	8	21.6
ER status of primary tumor	Positive	16	43.2
	Negative	21	56.8
PR status of primary tumor	Positive	13	35.1
	Negative	24	64.9
HER2 status of primary tumor	Positive	19	51.4
	Negative	18	48.6
Primary breast cancer subtype	HR+, HER2−	13	35.1
	HR+, HER2+	3	8.1
	HR−, HER2+	16	43.2
	HR−, HER2−	5	13.5
Multiplicity	Single brain metastasis	28	75.7
	Multiple metastasis	9	24.3
Brain metastasis location	Supratentorial	25	67.6
	Infratentorial	8	21.6
	Both	4	10.8

*ER* estrogen receptor, *PR* progesterone receptor, *HER2* human epidermal growth factor receptor 2, *HR* hormone receptor

**Fig. 1:**
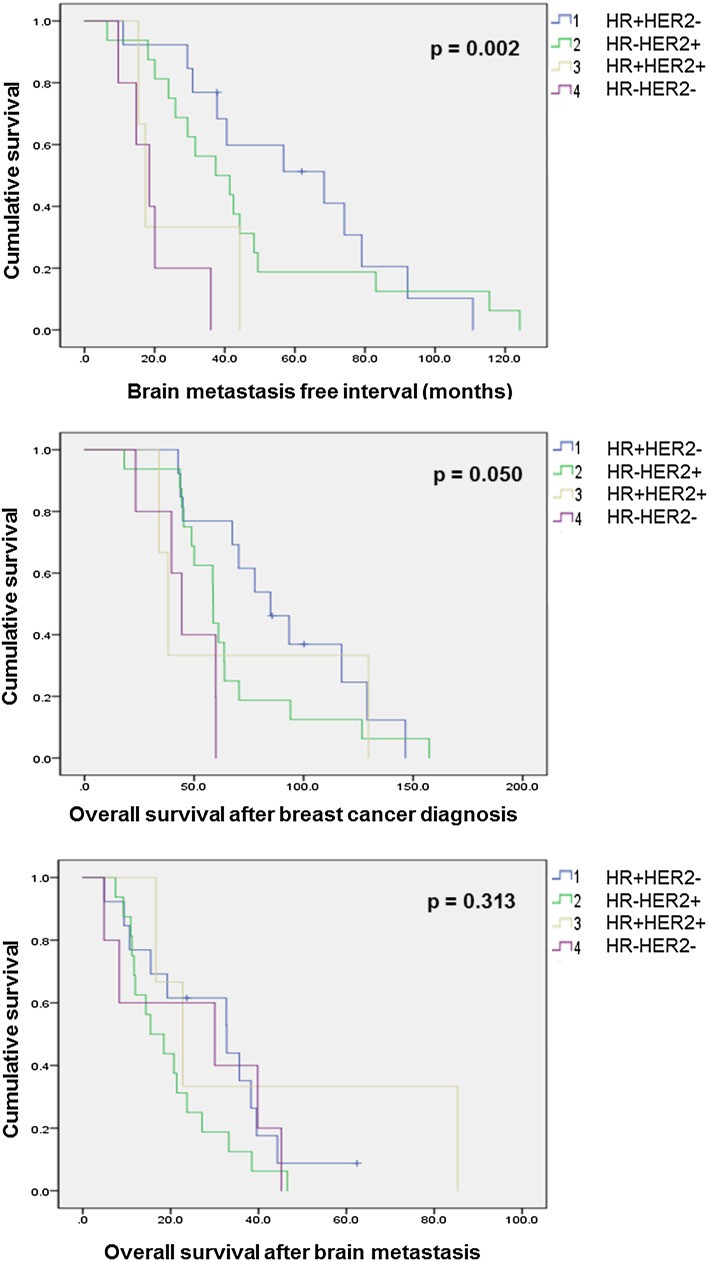
Brain metastasis free interval, overall survival after breast cancer diagnosis, and overall survival after brain metastasis according to breast cancer subtypes

Results of brain metastasis-free interval, overall survival after breast cancer diagnosis, and overall survival after brain metastasis according to breast cancer subtypes are shown in Fig. 1. Brain metastasis free interval and overall survival after breast cancer were statistically different between groups. However, overall survival after brain metastasis did not differ significantly between groups.

**Fig. 2:**
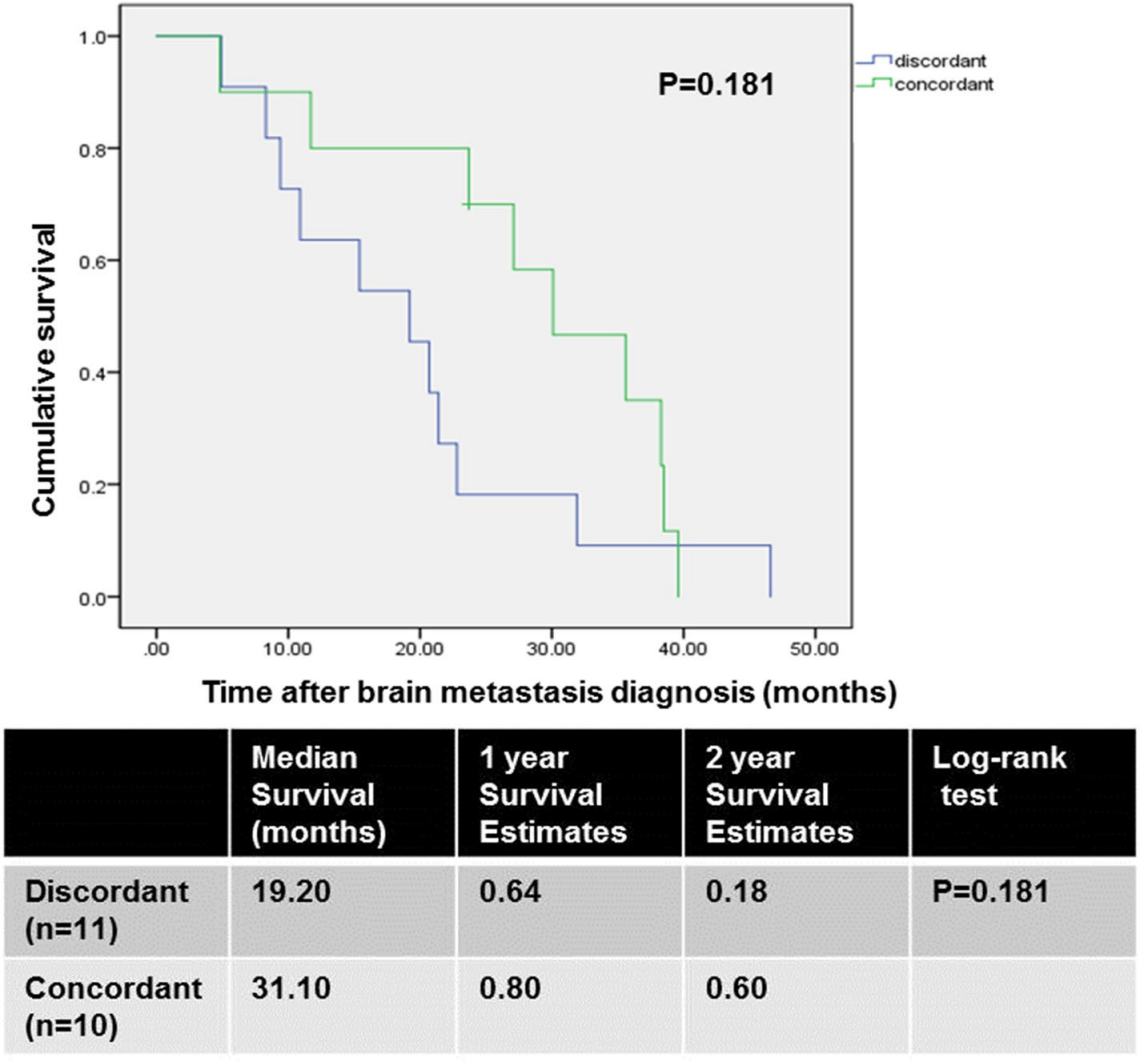
Overall survival comparison between the discordant and concordant groups

Among 37 patients, data for ER, PR, and HER2 expression in brain metastasis were available for 21 cases based on electronic medical records. Expressions of ER, PR, and HER2 in brain metastasis were positive in 47.6, 42.9 and 38.1% of patients, respectively. Receptor conversion occurred in 11 of 21 patients (52.4%): for ER, 9.5%; for PR, 38.1%; for HER2, 23.8% (Table 2). Overall survival was longer in patients with concordant receptor expression patterns between the primary breast cancer and brain lesion compared to that in patients with discordant patterns (Fig. 2). However, the difference was not statistically significant (discordant vs. concordant median survival: 19.2 vs. 31.1 months, *p* = 0.181). There was no statistically significant difference in median survival according to the conversion of each receptor (Table 3).

**Table 2:** ER, PR, and HER2 expression profiles in patients with primary breast cancer and brain metastasis

Brain metastasis	Primary breast cancer		Total
	**−**	+	
ER status			
**−**	10 (47.6%)	1 (4.8%)	11 (52.4%)
+	1 (4.8%)	9 (42.9%)	10 (47.6%)
PR status			
**−**	10 (47.6%)	2 (9.5%)	12 (57.1%)
+	6 (28.6%)	3 (14.3%)	9 (42.9%)
HER2 status			
**−**	10 (47.6%)	3 (14.3%)	13 (61.9%)
+	2 (9.5%)	6 (28.6%)	8 (38.1%)

*ER* estrogen receptor, *PR* progesterone receptor, *HER2* human epidermal growth factor receptor 2

**Table 3:** Median survival comparison between the discordant and concordant groups according to receptor profiles in patients with primary breast cancer and brain metastasis

	Median survival (months)	Log-rank test (p value)
ER conversion		0.853
Discordant (n = 2)	4.9	
Concordant (n = 19)	22.8	
PR conversion		0.310
Discordant (n = 8)	15.4	
Concordant (n = 13)	30.1	
HER2 conversion		0.389
Discordant (n = 5)	21.4	
Concordant (n = 16)	22.8	

*ER* estrogen receptor, *PR* progesterone receptor, *HER2* human epidermal growth factor receptor 2

## Discussion

Biomolecular markers are becoming the most important factors for systemic therapy of breast cancer patients such as hormonal therapy, chemotherapy, or targeted therapy. Studies performed in the last decade have revealed that receptor expression of metastatic lesions does not always reflect its status in primary tumor. To have more effective systemic therapy, molecular diagnosis through biopsy of metastatic lesions is strongly recommended. However, biopsies for brain metastases are not always performed in routine clinical practice due to limited access to metastatic brain lesions.

In terms of HER2, Niikura et al. [11]. have reported that the relatively frequent HER2 negative conversion rate is 24% in 182 patients with HER2-positive primary breast cancer and systemic metastasis. They have demonstrated that HER2 discordance is correlated with poorer survival. Therefore, they strongly recommend biopsies for metastatic lesions in primary HER2-positive breast cancer to obtain accurate molecular diagnosis and appropriate therapy. Lindstrom et al. [14] have also demonstrated that patients with HER2 discordant lesions have poorer survival. However, Amir et al. [15] have reported that HER2 discordance is not associated with detrimental effects on outcome.

There is no definite treatment guideline for BCBMs. The main goal is to alleviate symptoms when treating such tumors. If brain lesion is solitary or < 3 lesions, it can be removed by craniotomy or radiosurgery. However, for cases of multiple brain metastases, WBRT and systemic therapy should be administered.

Recent studies have shown that anti-HER2 treatment can improve survival after BCBM diagnosis [5, 6, 16]. It is currently unclear whether such improvement is due to extracranial disease control or idirect intracranial tumor response. However, it has been reported that HER2-targeting agent trastuzumab can penetrate the impaired blood–brain barrier at the site of metastasis [17]. Swain et al. [18] have reported that adding pertuzumab to docetaxel and trastuzumab can delay the onset of brain metastases. Krop et al. [19] have also reported that trastuzumab emtansine (T-DM1) is associated with significantly improved overall survival in patients with BCBM. Therefore, when response of anti-HER2 treatment is poor in patients with BCBM, identifying receptor expression patterns in brain metastases should be considered.

Because it is difficult to reach brain metastasis sites, such sites are not routinely biopsied. A few studies have reported receptor status conversion between primary tumors and brain metastases [4, 7, 9, 10, 20–22]. However, most of these studies included small groups of BCBM. Thus, the incidence of receptor discordance between primary and metastatic tumor sites has not been conclusively established. Moreover, clinical impacts of receptor discordance such as prognosis and survivals in BCBM remain unclear.

Through the literature review, over 280 matched cases of BCBM were collected. Average discordance rates were 21.6% (range 13.6–29.2%) for ER, 26.7% (range 4.2–44.4%) for PR, and 10.7% (range 2.3–19.0%) for HER2 (Table 4). In a study by Duchnowska et al. [10], discordance rates for ER, PR, and HER2 status in primary tumor and brain metastases have been analyzed. In 120 cases of matched BCBM, discordance rates of ER, PR, and HER2 were 29, 24, and 14%, respectively. However, receptor conversion showed no significant impact on survival.

**Table 4:** Systemic review of previous studies on receptor discordance between primary breast cancer and brain metastases

	Markers	No. of cases	Positive conversion	Negative conversion	Total
Gaedcke et al. [16]	ER	23	2	4	6 (26.1%)
	PR	23	2	3	5 (21.7%)
	HER2	23	2	1	1 (4.2%)
Yonomori et al. [17]	ER	24	2	2	4 (16.7%)
	PR	24	0	1	1 (4.2%)
	HER2	24	1	2	3 (12.5%)
Omoto et al. [9]	ER	21	2	2	4 (19.0%)
	PR	21	1	3	4 (19.0%)
	HER2	21	3	1	4 (19.0%)
Hoefnage et al. [7]	ER	44	NA	NA	6 (13.6%)
	PR	44	NA	NA	16 (36.4%)
	HER2	44	NA	NA	1 (2.3%)
Shao et al. [18]	ER	18	0	1	6 (16.2%)
	PR	18	4	4	8 (44.4%)
	HER2	18	1	0	1 (5.6%)
Brogi et al. [4]	ER	37	NA	NA	6 (16.2%)
	PR	39	NA	NA	8 (20.5%)
	HER2	40	0	2	2 (5.0%)
Duchnowska et al. [10]	ER	120	13	22	35 (29.2%)
	PR	119	11	18	29 (24.4%)
	HER2	119	10	7	17 (14.3%)
Total	ER	287			62 (21.6%)
	PR	288			71 (26.7%)
	HER2	289			31 (10.7%)

*ER* estrogen receptor, *PR* progesterone receptor, *HER2* human epidermal growth factor receptor 2, *NA* not applicable

In the present study, discordance rates of ER, PR, and HER2 status were 9.5, 38.1, and 23.8%, respectively. About half of these cases showed discordance for more than one receptor. Our results showed less frequent ER changes but higher rate of discordance in HER2 than those of other studies. Reasons for such differences are currently unclear. However, it is known that breast cancer in Korean women has different epidemiological features compared to breast cancer in women from other countries [1]. First, the incidence of breast cancer is lower in Korea than that in western countries. Second, women in western countries are more likely to have breast cancer as their age increases. However, the incidence of breast cancer in Korean women increases until early 50 s. It then gradually decreases thereafter. Third, the prevalence of breast cancer patients before menopause is much higher in Korea than that in western countries.

Any changes in receptor expression should be interpreted with caution as variation in processing of tissues can lead to incorrect results. Inadequate fixation can also result in false negatives or false positives. Although adequate fixation is achieved, receptor expression may be discordant due to intratumoral heterogeneity [23]. Changes in receptor expression might also occur due to tumor resistance to endocrine treatment [24] or HER2-targeted therapy [25].

This study has some limitations. First, there might be methodological and selection biases due to the retrospective nature of this study. Biopsy and IHC analyses of primary and metastatic lesions were not performed simultaneously. Moreover, subjects of this study were patients who underwent craniotomy. Clinicians generally recommend surgery only for patients with favorable prognostic factors. Second, the total number of patients included in this study was small. Although treatment options for BCBM were local (such as surgery), breast cancer patients generally did not opt for brain surgery readily. However, the fact that this study was conducted at a single institution was an advantage in this regard since the overall treatment policies for patients and the anticancer drugs used were uniform. In addition, this study was meaningful because it was the first study that compared receptor profiles between primary breast cancer lesions and brain metastases in Korean population.

In conclusion, receptor conversion in BCBMs occurred in about 50% of Korean BCBM patients included in this study. HER2 conversion was observed in 23.8% of these patients. Therefore, if the metastatic lesion is the brain alone, it is better to perform surgery than stereotactic radiosurgery because receptor profile of the distant metastatic site can be identified. Also, if resistance to anti-HER2 treatment is suspected in patients with BCBM, it should be considered the possibility that receptor conversion has occurred. Further studies are needed to determine whether differences in receptor expression levels between primary and metastatic brain lesions are prognostically relevant.
